# Supplementary material for: Development of an HRMA-Based Marker Assisted Selection (MAS) Approach for Cost-Effective Genotyping of S and M Loci Controlling Self-Compatibility in Apricot (Prunus armeniaca L.)
Source: Genes (Basel). 2022 Mar 20;13(3):548. doi: 10.3390/genes13030548 (PMC8954599; doi:10.3390/genes13030548)
Supplement: Supplementary file 1 [file genes-13-00548-s001.zip › Supplementary_files/Supplementary_File_1.pdf]

SFB sequences alignment

```
            421                                                          480
AY587563.1  GATTTCCTTG CCTATTACCA ATCTCAAAAG TGAGTCATTG TCATTGGACT TTCTGCAAAA
AY587562.1  CTTCCCATTG CCGATTACGA ACCTCAAAAG TGAGTCATTG ------GACT TTCTGCGAAA
AY587565.1  CTTTCCATTG CCCAT-ACCA ACCTCAGAAG TAAGTCATTG ------G--- TTCTGCAAAA
DQ422946.1  CCCTTCCTTG CCTATTCCCA ACCTCAAAAG TGAGTCGTTG ------GACT TTCTGCAAAA
DQ887488.1         TTG GCTATTRTCA ACCGCAAAAG TGAGTCGTTG ------GACT TTCTGCAAAA
EF101912.1
DQ897929.1         TTA GCTATTSTMA -CCTCAAAAG TGAGTCGTTG ------GACT TTCTGCAAAA
FJ477418.1         TTG CCTATTATCA ACCGCAAAAG TGAGTACT-- -----GCACT TTGTGCAAAA
EF053406.1
            481                                                          540
AY587563.1  ATGGAAGCAC CAATTTATTT CCTAACTTGA TTGTATTACA ATCCATATTC TAAATTCGAA
AY587562.1  AAGAAAGCAC AATTGT-TTT ---AACTTTG TTCCGTTATG ATCCATACTT TAGATTCTAA
AY587565.1  CTAAAAGCAC CATTGT-CTT CCTAACTTTG TTTTGCAACG ATCCATACTT TAGATTCTAA
DQ422946.1  ATGAAAAGCA CTATTGTTTT CC-----TTG TTTTGTTATG ATCAATATTT TGGATGCTAA
DQ887488.1  ATGAAAAGCA CTATTGTTTT CC-----TTG TTTTGTTATG ATCAATATTT TGGATGCTAA
EF101912.1
DQ897929.1  ATGAAAAGCA CTATTGTTTT CC-----TTG TTTTGTTATG ATCAATATTT TGGATGCTAA
FJ477418.1  ATGAAAGAGC --ATTGCTTT CCTAACTTTG TTTTGTTATG ATCCGTACTT TAGATTCTAA
EF053406.1
            541                                                          600
AY587563.1  CTTATCTTAT TA------TG ATTCAGGATG GCATTCACAC TACGCAAGAA AGAAATCTTA
AY587562.1  CTTATTTTCA TTATGATTTT CTTCAGGATG ACATTCACGC TGCATAAGAA AGAAATCTTA
AY587565.1  CATATTTTCG TTATGAGTTT CTTCAGCATG ACATTCACTC TATGTAAGAA AGAAATTTTA
DQ422946.1  CTTATTTTGA TTATGA-TTT CTTCAGGATG ACATTCACAC TACGTAAGAA AGAAATCTTA
DQ887488.1  CTTATTTTGA TTATGA-TTT CTTCAGGATG ACATTCACAC TACGTAAGAA AGAAATCTTA
EF101912.1                                                  TAAGAA AGAAATCTTA
DQ897929.1  CTTATTTTGA TTATGA-TTT CTTCAGGATG ACATTCACAC TACGTAAGAA AGAAATCTTA
FJ477418.1  CATATTTTCA TTATGATTTT CTTCAGGATG GCATTCACAC TACGTAAGAA AGAAATCTTA
EF053406.1
            601        SFBc-F                                            660
AY587563.1  GTCGACATCC TAGTAAGACT ACCTGCAAAA TCTCTCGTTC GGTTTCTGTG TACATGCAAG
AY587562.1  ATCGACATCC TAGTAAGACT ACCCGCAAAA TCCCTCGTTC GGTTTCTGTG TACATGCAAG
AY587565.1  GTCGACATCC TGGTAAGACT GCCTGCAAAA TCTCTCGTTC GGTTTATGTG TACATGTAAG
DQ422946.1  ATCGACATCC TAGTAAGACT ACCTGCAAAA TCCCTTATTC GCTTTCTGAG TACATGCAAG
DQ887488.1  ATCGACATCC TAGTAAGACT ACCTGCAAAA TCCCTTATTC GCTTTCTGAG TACATGCAAG
EF101912.1  ATCGACATCC TAGTAAGACT ACCTGCAAAA TCCCTTATTC GCTTTCTGAG TACATGCAAG
DQ897929.1  ATCGACATCC TAGTAAGACT ACCTGCAAAA TCCCTTATTC GCTTTCTGAG TACATGCAAG
FJ477418.1  ATCGACATCC TACTAAGACT ACCTGCAAAA TCCCTCATTC GGTTTCTAAG TACATGCAAG
EF053406.1                                 T TCTCTTGTTC GGTTTCTGTG TACATGCAAG
            661                                                          720
AY587563.1  TCATGGAGTG GTTTGATTGG CAGCTCGAGT TTTGTTAGCA TACACCTTAA CAGGAATGTC
AY587562.1  TTATGGGGTG ATTTGATTGG CAGCTCAAGT TTTGTTAGTG CACACCTTAA TAGGAATGTC
AY587565.1  TCATGGAGTG ATTTGATTGG CAGCTCCAGT TTTGTTAGCA CACACCTTAA TAGGAATGTC
DQ422946.1  TCGTGGAGTG ATTTGATTGG AAGCTCAATT TTTGTTAGCA CACACCTTTG TAGGAATGTG
DQ887488.1  TCGTGGAGTG ATTTGATTGG AAGCTCAATT TTTGTTAGCA CACACCTTTG TAGGAATGTG
EF101912.1  TCGTGGAGTG ATTTGATTGG AAGCTCAATT TTTGTTAGCA CACACCTTTG TAGGAATGTG
DQ897929.1  TCGTGGAGTG ATTTGATTGG AAGCTCAATT TTTGTTAGCA CACACCTTTG TAGGAATGTG
FJ477418.1  TCATGGAGTG ATTTGATTGG CAGCTCGAGT TTTGTTAGCA CACATCTTAA TAAGAATGTC
EF053406.1  TCGTGGAGTG ATTTGATTGG AAGCTCAATT TTTGTTAGCA CACACCTTTG TAGGAATGTG
            721                                                          780
AY587563.1  ACAGAACATG CCCATGTCTA TCTACTCTGC CTCCTCCACC CAAATTTTGA ACGTCTGGCC
AY587562.1  ACAAAACGTG CCCCTGTATA TCTTCTTTGC CTCCACCACC CAAATTTTGA ACGTCAGAAC
AY587565.1  AATAAACATG CCCATGTATA TCTACTTTGC CTCCACCATC CAAATTTTGA ATGTGTGGTG
DQ422946.1  ACAAAACATA CCCATGTTTA TCTACTTTGC CTCCACCACC CAAATTTTGA GCGTTTGGTC
DQ887488.1  ACAAAACATA CCCATGTTTA TCTACTTTGC CTCCACCACC CAAATTTTGA GCGTTTGGTC
```

```
EF101912.1    ACAAAACATA CCCATGTTTA TCTACTTTGC CTCCACCACC CAAATTTTGA GCGTTTGGTC
DQ897929.1    ACAAAACATA CCCATGTTTA TCTACTTTGC CTCCACCACC CAAATTTTGA GCGTTTGGTC
FJ477418.1    ACAAAACATG CCCATGTCTA TCTACTTTGC CTCCACCACC CAAATTTTGA ATGTGTGATC
EF053406.1    ACAAAACATA CCCATGTTTA TCTACTTTGC CTCCACCACC CAAATTTTGA GCGTTTGGTC
              781                                                           840
AY587563.1    GACCCTGATG ACCCATATGT TAAACAAGAG TTTAGCTGGT CTCTTTTTTC GAATGAAACA
AY587562.1    GACACTGATG ACCCATATGA CATAGAAGAA CTTCAGTGGT CACTTTTTTC CAAAGAAACA
AY587565.1    GACCCTGATG ACCCATATTT AGAAGAAGAA GTTCAATGGT CTCTTTTTCC CAATGAAACA
DQ422946.1    GACCCTAATG ACCCATATCT TAAAAAGGAA TTTCAATGGT CTCTTTTTCC CAAAGAAACA
DQ887488.1    GACCCTAATG ACCCATATCT TAAAAAGGAA TTTCAATGGT CTCTTTTTCC CAAAGAAACA
EF101912.1    GACCCTAATG ACCCATATCT TAAAAAGGAA TTTCAATGGT CTCTTTTTCC CAAAGAAACA
DQ897929.1    GACCCTAATG ACCCATATCT TAAAAAGGAA TTTCAATGGT CTCTTTTTCC CAAAGAAACA
FJ477418.1    GACCCCGATG ACCCATATTT AGAAGAAGAA CTTCAATGGT CTCTGTTTTC CAATGTAACA
EF053406.1    GACCCTAATG ACCCATATCT TAAAAAGGAA TTTCAATGGT CTCTTTTTCC CAAAGAAACA
              841        RFBc-F                                             900
AY587563.1    TTTGAGGAAA GCTCCAAGAT AACCCATCCC TTAGGGAGCA CAGAACATTA TGGGATATAT
AY587562.1    TTTGAGCAGT TCTCCAAGTT AACCCATCCC TTAGGAAGCA CAGAATATTA TGGGGTATAT
AY587565.1    TTTGAGGAAT GCTCCAAGTT AAACCATCCC TTAGGGAGCA CAGAACATTA TGGGATATAT
DQ422946.1    TTTGAGGAGT GCTACAAACT AAGCCATCCC TTAGGGATGA CAGAACATTA TGGGATATAT
DQ887488.1    TTTGAGGAGT GCTACAAACT AAGCCATCCC TTAGGGATGA CAGAACATTA TGGGATATAT
EF101912.1    TTTGAGGAGT GCTACAAACT AAGCCATCCC TTAGGGATGA CTGAACATTA TGGGATATAT
DQ897929.1    TTTGAGGAGT GCTACAAACT AAGCCATCCC TTAGGGATGA CAGAACATTA TGGGATATAT
FJ477418.1    TTTGAGAAGT GCTCCAAGTT AAGCCATCCC CTAGGAAGCA CAAAACATTA TGGGATATAT
EF053406.1    TTTGAGGAGT GCTACAAACT AAGCCATCCC TTAGGGATGA CAGAACATTA TGGGATATAT
              901                                                           960
AY587563.1    GGTTCAAGCA ATGGTTTAGT TTGCATTTCA GATGAGATTT TGAATTTCGA TAGTCCTATA
AY587562.1    GGTTCAAGCA ATGGTTTAGT TTGCATTTCG GATGAGATCT TGAATTTCGA TAGTCCTATA
AY587565.1    GGCTCCAGCA ATGGTTTAGT TTGCATTTCG GATGAGATAT TGAATTTCGA TAGTCCTATA
DQ422946.1    GGTTCAAGCA ATGGTTTAGT TTGCATTTCG GATGAGATCC TGAATTTCGA TAGTCCTATA
DQ887488.1    GGTTCAAGCA ATGGTTTAGT TTGCATTTCG GATGAGATCC TGAATTTCGA TAGTCCTATA
EF101912.1    GGTTCAAGCA ATGGTTTAGT TTGCATTTCG GATGAGATCC TGAATTTCGA TAGTCCTATA
DQ897929.1    GGTTCAAGCA ATGGTTTAGT TTGCATTTCG GATGAGATCC TGAATTTCGA TAGTCCTATA
FJ477418.1    GGTTCAAGCA ATGGTTTGCT TTGCATTTCT GATGAGATAT TGAATTTCGA TAGTCCAATA
EF053406.1    GGTTCAAGCA ATGGTTTAGT TTGCATTTCG GATGAGATCC TGAATTTCGA TAGTCCTATA
              961                                                          1020
AY587563.1    CACATATGGA ACCCGTCGGT TAAGAAATTT AAGACCCCTC CAATGAGCAC CAACATTAAC
AY587562.1    CACATATGGA ACCCATCAGT TAGGAAATTT AGGACCCCTC AAATGAGCAC CAACATTAAC
AY587565.1    CATATATGGA ACCCATCAGT TAGGAAACTT CGGACCCTTC CAATAAGCAC CAACATCA--
DQ422946.1    CACATATGGA ACCCATCGGT TAGGAAATTT AGGGCCCTTC CAATGAGCAC CAACATTAAC
DQ887488.1    CACATATGGA ACCCATCGGT TAGGAAATTT AGGGCCCTTC CAATGAGCAC CAACATTAAC
EF101912.1    CACATATGGA ACCCATCGGT TAGGAAATTT AGGGCCCTTC CAATGAGCAC CAACATTAAC
DQ897929.1    CACATATGGA ACCCATCGGT TAGGAAATTT AGGGCCCTTC CAATGAGCAC CAACATTAAC
FJ477418.1    CACATATGGA ACCCATTGGT TGGAAGATAT AGGACCCCTC CAATGAGCAC CAACATTAAC
EF053406.1    CACATATGGA ACCCATCGGT TAGGAAATTT AGGGCCCTTC CAATGAGCAC CAACATTAAC
              1021                                                         1080
AY587563.1    ATTAAATTTA GCCTCGTTTC TCTCCAATTC GGGTTCCATC CTAGGGTTAA TGACTACAAG
AY587562.1    GTTAAATTTA GCTATGTTGC TCTCCAATTC GGGTTCCACC CCCTCGTTAA TGACTACAAG
AY587565.1    -TTAAATTTA GCCATGTTGC TCTCCAGTTC GGGTTCCATC CCGGGGTTAA TGACTATAAG
DQ422946.1    ATTAAATTTA GCTGTGTTGC TCTCCAGTTC GGGTTCCACC CTGGGGTTAA TGACTACAAG
DQ887488.1    ATTAAATTTA GCTGTGTTGC TCTCCAGTTC GGGTTCCACC CTGGGGTTAA TGACTACAAG
EF101912.1    ATTAAATTTA GCTGTGTTGC TCTCCAGTTC GGGTTCCACC CTGGGGTTAA TGACTACAAG
DQ897929.1    ATTAAATTTA GCTGTGTTGC TCTCCAGTTC GGGTTCCACC CTGGGGTTAA TGACTACAAG
FJ477418.1    ATTAAATTTA ATTATGTTGC TCTCCAATTC GGGTTTCACC CTGGGGTTAA TGACTACAAG
EF053406.1    ATTAAATTTA GCTGTGTTGC TCTCCAGTTC GGGTTCCACC CTGGGGTTAA TGACTACAAG
              1081                                                         1140
AY587563.1    GCTGTAAGGA TGATGCGCAC CAACAAAAAT GTCTTGGCGG TTGAGGTTTA TAGTCTCAGC
AY587562.1    GCTGTAAGGA TGATGCGTAC CAACAAAAAT TCTTTGGCAG TTGAGGTTTA TAGTCTTAGA
```

```
AY587565.1  GCTGTAAGGA TGATGCGTAC CAACAAAAAT GCCTTGGCGA TTGAGGTGTA TAGTCTTAGA
DQ422946.1  GCTGTAAGGA TGATGCGTAC CAATAAAAGT GCCTTGGCGG TTGAGGTTTA TAGTCTCAAA
DQ887488.1  GCTGTAAGGA TGATGCGTAC CAATAAAAGT GCCTTGGCGG TTGAGGTTTA TAGTCTCAAA
EF101912.1  GCTGTAAGGA TGATGCGTAC CAATAAAAGT GCCTTGGCGG TTGAGGTTTA TAGTCTCAAA
DQ897929.1  GCTGTAAGGA TGATGCGTAC CAATAAAAGT GCCTTGGCGG TTGAGGTTTA TAGTCTCAAA
FJ477418.1  GCTGTAAGGA TGATGCGTAC CAACAAAGAT GCCTTGGCGG TTGAGGTTTA TAGTCTTAGA
EF053406.1  GCTGTAAGGA TGATGCGTAC CAATAAAAGT GCCTTGGCGG TTGAGGTTTA TAGTCTCAAA
            1141                                                RFBc-R 1200
AY587563.1  ACAGACTCTT GGAAAATGGT TGAAGCAATT CCTCCTTGGT TAAAATGCAC TTGGCAGCAT
AY587562.1  ACAAACTCTT GGAAGATGAT TGAAGCAATT CCTCCTTGGT TGAAATGCAC TTGGCAGCAT
AY587565.1  ACAGACTCTT GGAAGATGAT TGAAGCAATT CCTCCTTGGT TAAAGTGCGC TTGGCAGCAT
DQ422946.1  AGAGACTCTT GGAAGATGAT TGAAGCAATT CCTCCTTGGT TAAAATGCAC TTTGGAACAT
DQ887488.1  AGAGACTCTT GGAAGATGAT TGAAGCAATT CCTCCTTGGT TAAAATGCAC TTTGGAACAT
EF101912.1  AGAGACTCTT GGAAGATGAT TGAAGCAATT CCTCCTTGGT TAAAATGCAC TTTGGAACAT
DQ897929.1  AGAGACTCTT GGAAGATGAT TGAAGCAATT CCTCCTTGGT TAAAATGCAC TTTGGAACAT
FJ477418.1  ACAGACTCGT GGAAGATGAT TGAAGCAATT CCTCCTTGGT TAAAATGCAC TTGGCAGCAT
EF053406.1  AGAGACTCTT GGAAGATGAT TGAAGCAATT CCTCCTTGGT TAAAATGCAC TTTGGAACAT
            1201                         SFBj-F                         1260
AY587563.1  CATAAGGGTA CATTTTTTAA TGGAGTAGCA TACCACATCA TTCAGAAAGG TCCTCTATTC
AY587562.1  TATAAGGGTA CCTTTTTTAA TGGAGTAGCA TACCACATCA TCGAGAAAGG TCCTATATTC
AY587565.1  TATAAGGGTA CATTTTTCAA TGGAGTAGCA TACCATATCA TTGAAAAAGG TCCTATATTC
DQ422946.1  CATAGGGGTA CGTTTTTCAG TGGAGTAGCA TACCACATCA TTCAGAAAGG TCCTATGCTC
DQ887488.1  CATAGGGGTA CGTTTTTCAG TGGAGTAGCA TACCACATCA TTCAGAAAGG TCCTATGCTC
EF101912.1  CATAGGGGTA CGTTTTTCAG TGGAGTAGCA TACCACATCA TTCAGAAAGG TCCTATGCTC
DQ897929.1  CATAGGGGTA CGTTTTTCAG TGGAGTAGCA TACCACATCA TTCAGAAAGG TCCTATGCTC
FJ477418.1  CATATGGGTA CATTTTTTAA TGGAGTAGCA TACCATATTA TTGAGAAAGG TCCCATATTC
EF053406.1  CATAGGGGTA CGTTTTTCAG TGGAGTAGCA TACCACATCA TTCAGAAAGG TCCTATGCTC
            1261                    SFBc-R                              1320
AY587563.1  AGCATTATGT CCTTCGATTC AGGCAGTGAA GAATTCGAAG AATTCATAGC ACCAGATGCC
AY587562.1  AGCATTATGT CCTTTGATTC AGGGAGTGAA GAATTCGAAG AATTCATAGC ACCAGATGCC
AY587565.1  AGCATCATGT CCTTCGATTC AGGCAGTGAA GAATTTGAGG AATTCATAGC ACCAGATGCA
DQ422946.1  AGCATTATGT CATTCGATTC AGGCAGTGAA AAATTCGAAG AAATCATAGC ACCAGATGCC
DQ887488.1  AGCATTATGT CATTCGATTC AGGCAGTGAA AAATTCGAAG AAATCATAGC ACCAGATGCC
EF101912.1  AGCATTATGT CATTCGATTC AGGCAGTGAA AAATTCGAAG AAATCATAGC ACCAGATGCC
DQ897929.1  AGCATTATGT CATTCGATTC AGGCAGTGAA AAATTCGAAG AAATCATAGC ACCAGATGCC
FJ477418.1  AGCATTATGT CCTTCGATTC AAGCAGTGAA GAATTTGAAG AGTTCATAGC ACCAGATGCC
EF053406.1  AGCATTATGT CATTCGATTC AGGCAGTGAA AAATTCGAAG AAATCATAGC ACCAGATGCC
            1321                                                        1380
AY587563.1  ATTTGCCGTT CATTGGGGTT ATGCATCGAC GTTTACAAGG AACACATTTG CTTGCTTTTT
AY587562.1  ATTTGCAGTT CATGGGGGTT ATGTATTGAC GTTTACAAGG AAAATATTTG CTTGCTTCTT
AY587565.1  ATTTGCCGTC CATCTGAGGT ATGTATTGAC GTTTACAAGG AACAAATTTG CTTGCTTTTT
DQ422946.1  ATTTGCAGTT TATGGGGGTT ATATATTGAC GTTTACAAGG AACAAATTTG CTTGCTTTTT
DQ887488.1  ATTTGCAGTT TATGGGGGTT ATATATTGAC GTTTACAAGG AACAAATTTG CTTGCTTTTT
EF101912.1  ATTTGCAGTT TATGGGGGTT ATATATTGAC GTTTACAAGG AACAAATTTG CTTGCTTTTT
DQ897929.1  ATTTGCAGTT TATGGGGGTT ATATATTGAC GTTTACAAGG AACAAATTTG CTTGCTTTTT
FJ477418.1  ATTTGCAGTT CATGGAGGTT ATGCATCAGC GTTTACAAGG AACAACTTTG CTTGCTTTTT
EF053406.1  ATTTGCAGTT TATGGGGGTT ATATATTGAC GTTTACAAGG AACAAATTTG CTTGCTTTTT
            1381                                                SFB-F  1440
AY587563.1  GGATTTTATG GTTGTGAGGA GGAGGGCATG GACAAAATTG ACTTATGGGT TCTGCAAGAG
AY587562.1  CAGTTTTATT CTAGTGAGGA GGAGGGGATG CGAAAAGCTG ACTTATGGGT TCTGCAAGAA
AY587565.1  GGATTTTATC GTTGTGAGGA GATGGGCATG GATAAAATTG ACTTGTGGGT TCTGCAAGAA
DQ422946.1  ATATGTTATG GGTGTGAGGA GGAGGGCATG GAAAAAGCTG ACTTATGGGT TCTGCAAGAA
DQ887488.1  ATATGTTATG GGTGTGAGGA GGAGGGCATG GAAAAAGCTG ACTTATGGGT TCTGCAAGAA
EF101912.1  ATATGTTATG GGTGTGAGGA GGAGGGCATG GAAAAAGCTG ACTTATGGGT TCTGCAAGAA
DQ897929.1  ATATGTTATG GGTGTGAGGA GGAGGGCATG GAAAAAGCTG ACTTATGGGT TCTGCAAGAA
FJ477418.1  GGATTTTATG GTTGTGAGGA GGAGGACATG GAAAAACTTC TCTTATGGGT TCTGCAAGAA
EF053406.1  ATATGTTATG GGTGTGAGGA GGAGGGCATG GAAAAAGCTG ACTTATGGGT TCTGCAAGAA
```

```
               1441                            SFBcj-F                               1500
AY587563.1     AAACGGTGGA AAAAATTGTG TCCTTTTATT TA-------- ---------- ----------
AY587562.1     AAACGGTGGA AGCAATTGTG TCCTTTTATT TA-------- ---------- ----------
AY587565.1     AAACGGTGGA AACAGTTGTG TCCTTTTATT TA-------- ---------- ----------
DQ422946.1     AAACGGTGGA AACAATTGAG TCCTTTTATT TAGAGATATT TAGTGATATA CCCATTCTTA
DQ887488.1     AAACGGTGGA AACAATTGAG TCCTTTTATT TAGAGATATT TAGTGATATA CCCATTCTTA
EF101912.1     AAACGGTGGA AACAATTGAG TCCTTTTATT TAGAGATATT TAGTGATATA CCCATTCTTA
DQ897929.1     AAACGGTGGA AACAATTGAG TCCTTTTATT TA-------- ---------- ----------
FJ477418.1     AAACGGTGGA AACAATTGTG TCCTGTTATT TA-------- ---------- ----------
EF053406.1     AAACGGTGGA AACAATTGAG TCCTTTTATT TA-------- ---------- ----------
               1501                                                                  1560
AY587563.1     ---------- ---------- ---------- ---------- ---------- ----------
AY587562.1     ---------- ---------- ---------- ---------- ---------- ----------
AY587565.1     ---------- ---------- ---------- ---------- ---------- ----------
DQ422946.1     ACACTAATAT TATAGATAAA CCCTCCATCA TTGAATTTCT ATAAACAAAC CCAAAAAAAA
DQ887488.1     ACACTAATAT TATAGATAAA CCCTCCATCA TTGAATTTCT ATAAACAAAC CCAAAAAAAA
EF101912.1     ACACTAATAT TATAGATAAA CCCTCCATCA TTGAATTTCT ATAAACAAAC CCAAAAAAAA
DQ897929.1     ---------- ---------- ---------- ---------- ---------- ----------
FJ477418.1     ---------- ---------- ---------- ---------- ---------- ----------
EF053406.1     ---------- ---------- ---------- ---------- ---------- ----------
               1561                                                                  1620
AY587563.1     ---------- ---------- ---------- ---------- ---------- ----------
AY587562.1     ---------- ---------- ---------- ---------- ---------- ----------
AY587565.1     ---------- ---------- ---------- ---------- ---------- ----------
DQ422946.1     CCCAAAAAAT GACAACTGGC CTTATTGAAT TTAATATTGA TTATTAAATT ACTTTAATGC
DQ887488.1     CCCAAAAAAT GACAACTGGC CTTATTGAAT TTAATATTGA TTATTAAATT ACTTTAATGC
EF101912.1     CCCAAAAAAT GACAACTGGC CTTATTGAAT CTAATATTGA TTATTAAATT ACTTTAATGC
DQ897929.1     ---------- ---------- ---------- ---------- ---------- ----------
FJ477418.1     ---------- ---------- ---------- ---------- ---------- ----------
EF053406.1     ---------- ---------- ---------- ---------- ---------- ----------
               1621                        SFB-INS-F    SFB-INS-R                    1680
AY587563.1     ---------- ---------- ---------- ---------- ---------- ----------
AY587562.1     ---------- ---------- ---------- ---------- ---------- ----------
AY587565.1     ---------- ---------- ---------- ---------- ---------- ----------
DQ422946.1     CCTATTGAGT GTTTTGGGTA TTTTTATGAG ATTTTGGGGT TGGGCTTGTT TTAAGAAATT
DQ887488.1     CCTATTGAGT GTTTTGGGTA TTTTTATGAG ATTTTGGGGT TGGGCTTGTT TTAAGAAATT
EF101912.1     CCTATTGAGT GTTTTGGGTA TTTTTATGAG ATTTTGGGGT TGGGCTTGTT TTAAGAAATT
DQ897929.1     ---------- ---------- ---------- ---------- ---------- ----------
FJ477418.1     ---------- ---------- ---------- ---------- ---------- ----------
EF053406.1     ---------- ---------- ---------- ---------- ---------- ----------
               1681                                                                  1740
AY587563.1     ---------- ---------- ---------- ---------- ---------- ----------
AY587562.1     ---------- ---------- ---------- ---------- ---------- ----------
AY587565.1     ---------- ---------- ---------- ---------- ---------- ----------
DQ422946.1     TATGGTAGTT TTGTAATTTC TAAGAAGCTA AAGCCTTTTT GTTATATTGT AAATGGGTTT
DQ887488.1     TATGGTAGTT TTGTAATTTC TAAGAAGTTA AAGCCTTTTT GTTATATTGT AAATGGGTTT
EF101912.1     TATGGTAGTT TTGTAATTTC TAAGAAGTTA AAGCCTTTTT GTTATATTGT AAATGGGTTT
DQ897929.1     ---------- ---------- ---------- ---------- ---------- ----------
FJ477418.1     ---------- ---------- ---------- ---------- ---------- ----------
EF053406.1     ---------- ---------- ---------- ---------- ---------- ----------
               1741                                                                  1800
AY587563.1     ---------- ---------- ---------- ---------- ---------- ----------
AY587562.1     ---------- ---------- ---------- ---------- ---------- ----------
AY587565.1     ---------- ---------- ---------- ---------- ---------- ----------
DQ422946.1     TGGGTGTGTT TATAAAGTTC ATTTCATATA GGGTATTTTT ATAATTTGGG TCTTCATATT
DQ887488.1     TGGGTGTGTT TATAAAGTTC ATTTCATATA GGGTATTTTT ATAATTTGGG TCTTCATATT
EF101912.1     TGGGTGTGTT TATAAAGTTC ATTTCATATA GGGTATTTTT ATAATTTGGG TCTTCATATT
```

```
DQ897929.1  ---------- ---------- ---------- ---------- ---------- ----------
FJ477418.1  ---------- ---------- ---------- ---------- ---------- ----------
EF053406.1  ---------- ---------- ---------- ---------- ---------- ----------
            1801                        SFBcj-R                       1860
AY587563.1  ---------- ---------- -------TGA TCCTCTGGAT TATTGTTATC GTATAATTGG
AY587562.1  ---------- ---------- ---------- TCCTTT---T CATTATTATA GTCCAATCGG
AY587565.1  ---------- ---------- ---------- TCCATGGGGT TTTTGTTATC GTATAACCGG
DQ422946.1  GGGTATATTA GTAAATCTCC CTTTTATTTA TCCTCCGGAT TATTATTATT GTACAACAGG
DQ887488.1  GGGTATATTA GTAAATCTCC CTTTTATTTA TCCTCCGGAT TATTATTATT GTACAACAGG
EF101912.1  GGGTATATTA GTAAATCTCC CTTTTATTTA TCCTCCGGAT TATTATTATT GTACAACAGG
DQ897929.1  ---------- ---------- ---------- TCCTCCGGAT TATTATTATT GTACAACAGG
FJ477418.1  ---------- ---------- ---------- TCCTGAAGGT AATTGTCGTC ATATTCTCGG
EF053406.1  ---------- ---------- ---------- TCCTCCGGAT TATTATTATT GTACAACAGG
            1861                                                      1920
AY587563.1  GATTGGTATA GATAATGAAC TCTTAATGGA AACAGAAGAT TTCCTTAGGG GCGTAGGATA
AY587562.1  TTTTAGTATA GATAATGAAC TCTTAGTGGA ACGAAAAGAT TTCGCTAGGG GCATAGGGGA
AY587565.1  GATTAGTATA GATAATGAAC TCTTAATGGG AAGAAGAGAT TTCTTAAAGG GCGTAGCAGA
DQ422946.1  GATTAGTATG GATAACAAAA TCTTAATGCT AAGAGTAGAT GACATTAGAG GCATAAGAAA
DQ887488.1  GATTAGTATG GATAACAAAA TCTTAATGCT AAGAGTAGAT GACATTAGAG GCATAAGAAA
EF101912.1  GATTAGTATG GATAACAAAA TCTTAATGCT AAGAGTAGAT GACATTAGAG GCATAAGAAA
DQ897929.1  GATTAGTATG GATAACAAAA TCTTAATGCT AAGAGTAGAT GACATTAGAG GCATAAGAAA
FJ477418.1  AATTAGTATA GATAATGAAC TCTTAATGAC AGAAAGAGGT TTCGATACCG GCATAGCAGA
EF053406.1  GATTAGTATG GATAACAAAA TCTTAATGCT AAGAGTAGAT GACATTAGAG GCATAAGAAA
            1921                             SFB-OUT-R                1980
AY587563.1  TCTGCATTTG TGTAATAATG AATCCAAGCA AGTTCTTGAA ACAGGAATTG AGTTGGCTGT
AY587562.1  TCTGCATCTG TGCAACGACA AATCCAAGCA AGTCCTTGAA ACAGGAATTG AGTTAGCAGC
AY587565.1  TCTCTATTTG TGTAAGTACG AATCCAAGCA GGTTCTTGAA ACAGGAATTA AGTTGGGCAT
DQ422946.1  TCTGGATTTA TGTGATTACG AATCCAACCA AGTTCTTGAA ACAGGAATTG AGTTGGCCAC
DQ887488.1  TCTGGATTTA TGTGATTACG AATCCAACCA AGTTCTTGAA ACAGGAATTG AGTTGGCCAC
EF101912.1  TCTGGATTTA TGTGATTACG AATCCAACCA AGTTCTTGAA ACAGGAATTG AGTTGGCCAC
DQ897929.1  TCTGGATTTA TGTGATTACG AATCCAACCA AGTTCTTGAA ACAGGAATTG AGTTGGCCAC
FJ477418.1  TCTGTATCTG CGTAATTATG AATCCAAGCA AGTTCTTGAA ACAGGAATTA AGTTGGCCGT
EF053406.1  TCTGGATTTA TGTGATTACG AATCCAACCA AGTTCTTGAA ACAGGAATTG AGTTGGCCAC
            1981                                                      2040
AY587563.1  CATGAAATAT GGCGAAATCG AATTCTCTTA CACAATTACT TACATAGAAA GCTTGGTTTT
AY587562.1  CATGAAATAT GGCAAAATCG AATTCTTGTT TGTAATTACT TACATAGAAA GTATGGTTTT
AY587565.1  CATGACATAT GGGGAAATCG AATTCTTGTT TTCATTTACT TACATGGAGA GCTTGGTTTT
DQ422946.1  CATGAAATAT GGCGAAATCG AATTCTTGTT TTCAATTACT TACGTAGAAA GTTTGGTTTT
DQ887488.1  CATGAAATAT GGCGAAATCG AATTCTTGTT TTCAATTACT TACGTAGAAA CTTTACSCTT
EF101912.1  CATGAAATAT GGCGAAATCG AATTCTTGTT TTCAATTACT TACGTAGAAA GTTTGGTTTT
DQ897929.1  CATGAAATAT GGCGAAATCG AATTCTTGTT TTCAATTACT TACGTAGAAA CTTTACCTTT
FJ477418.1  CTTGAGATAT GGCGATATCG AATTCTTGTT TGCAATCACT TACATAGAAA GTTTGGTTTT
EF053406.1  CATGAAATAT GGCGAAATCG AATTCTTGTT TTCAATTACT TACGTAGAGA GTATGGTTTT
            2041                                                      2100
AY587563.1  TCTCGATAAA TATTAAATAG ATTTATAAAA TTTG--TCTC TTTTTTTTGG -----GATTT
AY587562.1  ACTCAATTAT TATTAAACAG AGGTATATAT AGTTAATTTT GTTTTCTGTC -----ATTTT
AY587565.1  ACTCAATTAA TTAGTAACAT ATATATAAAG T-----TTGG TTTTTCTTCC -----GCTTT
DQ422946.1  ACTCAATAAT TATTAAACAG ATGTATAAAG TTTGTTGTTA TTTGTTTTTC TTTCGGTTTA
DQ887488.1  ACTCAATA
EF101912.1  ACTCAATA
DQ897929.1  ACTCAATT
FJ477418.1  ACTCAACAAT TGTTAA
EF053406.1  ACTCAATA
```

ParMDO sequence alignment

```
            1                                                           60
```

```
KY429941  CGGATATCTA ATATGTCGCT AGACTCTGAA TCAAGATCGC ATATTCAAAT ATGATTTCAC
KY429940  CGGATATCTA ATATGTCGCT AGACTCTGAA TCAAGATCGC ATATTCAAAT ATGATTTCAC
KY499716
          61                                                           120
KY429941  TTAATCAATG ATTCCGATTA CACTCTAATA ACACCACATA CAAAATTGTT TAACAAAAGG
KY429940  TTAATCAATG ATTCCGATTA CACTCTAATA ACACCACATA CAAAATTGTT TAACAAAAGG
KY499716
          121                                                          180
KY429941  AAATTACTCA GCATTTAAAA AATATTTCCA AGGTCTGCAT TCTTATTGGA GCTGGGGAGT
KY429940  AAATTACTCA GCATTTAAAA AATATTTCCA AGGTCTGCAT TCTTATTGGA GCTGGGGAGT
KY499716
          181                                                          240
KY429941  GAATGCTTTA AGATGTGAAG TTGTATCATT TTGTAGCTGC TTCAAAAGCA GCTAGGAAGA
KY429940  GAATGCTTTA AGATGTGAAG TTGTATCATT TTGTAGCTGC TTCAAAAGCA GCTAGGAAGA
KY499716                            TCATT TTGTAGCTGC TTCAAAAGCA GCTAGGAAGA
          241                                                          300
KY429941  CCTCAGTTGG TTGGGCACCA GCAAATTCTT CCTTTCCATT AATCTGCCAA AAGAACCAAA
KY429940  CCTCAGTTGG TTGGGCACCA GCAAATTCTT CCTTTCCATT AATCTGCCAA AAGAACCAAA
KY499716  CCTCAGTTGG TTGGGCACCA GCAAATTCTT CCTTTCCATT AATCTGCCAA AAGAACCAAA
          301                                                          360
KY429941  TGATTAGTAG TGATGGATCT ATTTGGCTAA TCAAACAAAA CCCAAATTTT GAAAAGAAAA
KY429940  TGATTAGTAG TGATGGATCT ATTTGGCTAA TCAAACAAAA CCCAAATTTT GAAAAGAAAA
KY499716  TGATTAGTAG TGATGGATCT ATTTGGCTAA TCAAACAAAA CCCAAATTTT GAAAAGAAAA
          361                                                          420
KY429941  AAAGAAGTGA GTTTTCATCA CCCACCACAA AATATGGGAT TCCTTTTACT TCTGAGTACT
KY429940  AAAGAAGTGA GTTTTCATCA CCCACCACAA AATATGGGAT TCCTTTTACT TCTGAGTACT
KY499716  AAAGAAGTGA GTTTTCATCA CCCACCACAA AATATGGGAT TCCTTTTACT TCTGAGTACT
          421                                                          480
KY429941  TCTTAAGCTC TTCCTTGACC TTCATTGCAT CATAATGAGC GAGGTCAGTT CCACTCAAAA
KY429940  TCTTAAGCTC TTCCTTGACC TTCATTGCAT CATAATGAGC GAGGTCAGTT CCACTCAAAA
KY499716  TCTTAAGCTC TTCCTTGACC TTCATTGCAT CATAATGAGC GAGGTCAGTT CCACTCAAAA
          481                                                          540
KY429941  ATATATAGAA GAAAGTTTGG GGTTTGGTTT ATTTCTGAGA AAGGAATGAC TGTTGCAGGA
KY429940  ATATATAGAA GAAAGTTTGG GGTTTGGTTT ATTTCTGAGA AAGGAATGAC TGTTGCAGGA
KY499716  ATATATAGAA GAAAGTTTGG GGTTTGGTTT ATTTCTGAGA AAGGAATGAC TGTTGCAGGA
          541                                                          600
KY429941  TTCTAGTTTT AATTTGGCCT GTTTTGGAAC CAAAGGATCA TAACAAAAAT TCCACAATCC
KY429940  TTCTAGTTTT AATTTGGCCT GTTTTGGAAC CAAAGGATCA TAACAAAAAT TCCACAATCC
KY499716  TTCTAGTTTT AATTTGGCCT GTTTTGGAAC CAAAGGATCA TAACAAAAAT TCCACAATCC
          601                                                          660
KY429941  TCTATCTGGT TTTTGCTGTC CTTCAACATA GCTTGGCCTG GAAAATAATA GGAACATGAA
KY429940  TCTATCTGGT TTTTGCTGTC CTTCAACATA GCTTGGCCTG GAAAATAATA GGAACATGAA
KY499716  TCTATCTGGT TTTTGCTGTC CTTCAACATA GCTTGGCCTG GAAAATAATA GGAACATGAA
          661                                                          720
KY429941  GGAATTGGTG AAGAACAGAC CTCAGTGAGC CCATTGTTGG GGTCATCAAG AAACTCTGCT
KY429940  GGAATTGGTG AAGAACAGAC CTCAGTGAGC CCATTGTTGG GGTCATCAAG AAACTCTGCT
KY499716  GGAATTGGTG AAGAACAGAC CTCAGTGAGC CCATTGTTGG GGTCATCAAG AAACTCTGCT
          721                                                          780
KY429941  GCCCCTTCTA TTTCAATCTT TTCAGCACAT TTCAGAAGAT AATCCCTGCA CCCAAATTGC
KY429940  GCCCCTTCTA TTTCAATCTT TTCAGCACAT TTCAGAAGAT AATCCCTGCA CCCAAATTGC
KY499716  GCCCCTTCTA TTTCAATCTT TTCAGCACAT TTCAGAAGAT AATCCCTGCA CCCAAATTGC
          781                                                          840
KY429941  AAATGTTTGT GCCTCTTTTA AGTTTTAAAT TAGATTAAGG ATGTAAAAGT ACTTAACCCA
KY429940  AAATGTTTGT GCCTCTTTTA AGTTTTAAAT TAGATTAAGG ATGTAAAAGT ACTTAACCCA
KY499716  AAATGTTTGT GCCTCTTTTA AGTTTTAAAT TAGATTAAGG ATGTAAAAGT ACTTAACCCA
          841                                                          900
KY429941  GAACATATCT TTGCATCCAT AGCCATTGCT TGAAAATTAA GAATAGATTA ATCAATCCTC
```

```
KY429940  GAACATATCT TTGCATCCAT AGCCATTGCT TGAAAATTAA GAATAGATTA ATCAATCCTC
KY499716  GAACATATCT TTGCATCCAT AGCCATTGCT TGAAAATTAA GAATAGATTA ATCAATCCTC
          901                                                          960
KY429941  CCCATGTTAC AAAACTAAAC AAGAGAATAA ATCACCATAT GCAATCTTAC CTGTCCCCAA
KY429940  CCCATGTTAC AAAACTAAAC AAGAGAATAA ATCACCATAT GCAATCTTAC CTGTCCCCAA
KY499716  CCCATGTTAC AAAACTAAAC AAGAGAATAA ATCACCATAT GCAATCTTAC CTGTCCCCAA
          961                                                         1020
KY429941  TGAATCTCCC ATCTGTGAAA TATCCAAGAC AGATCTCATC CACTAGATCA TGCTGCTTAT
KY429940  TGAATCTCCC ATCTGTGAAA TATCCAAGAC AGATCTCATC CACTAGATCA TGCTGCTTAT
KY499716  TGAATCTCCC ATCTGTGAAA TATCCAAGAC AGATCTCATC CACTAGATCA TGCTGCTTAT
          1021                                                        1080
KY429941  CATGATCCTG TAGCCCAGCA AAATATATAT GGGAGATTCA CTAATATACC CAATATATGA
KY429940  CATGATCCTG TAGCCCAGCA AAATATATA- ---------- ---------- ----------
KY499716  CATGATCCTG TAGCCCAGCA AAATATATA- ---------- ---------- ----------
          1081                                                        1140
KY429941  GCCCAAATTA TAAAAAAACC CTATATGAAA TGGAATTTAT AAATACACCC AAAGCCCATT
KY429940  ---------- ---------- ---------- ---------- ---------- ----------
KY499716  ---------- ---------- ---------- ---------- ---------- ----------
          1141                                                        1200
KY429941  TATAACATAA CAAAAAAGCT TTTAACTTCT TTTAAATTAC AAAATTGCCA TTGATTTCTT
KY429940  ---------- ---------- ---------- ---------- ---------- ----------
KY499716  ---------- ---------- ---------- ---------- ---------- ----------
          1201                                                        1260
KY429941  AAAACAAACC CAACCCAAAA TTTCATAAAA AAACCCAAAA CACTCAATAG GGTATCAAAG
KY429940  ---------- ---------- ---------- ---------- ---------- ----------
KY499716  ---------- ---------- ---------- ---------- ---------- ----------
          1261                                                        1320
KY429941  TAATTTAATA ATTAAAATTA AATTTAATAG TACAATTTGT CATTTTTTGG GTTTTTTTTG
KY429940  ---------- ---------- ---------- ---------- ---------- ----------
KY499716  ---------- ---------- ---------- ---------- ---------- ----------
          1321                                                        1380
KY429941  GGTATGTTTA TAAAAATGAA ATGATGTAGG GTTTATTTAT AGTTTTAGTG CTAAGAATGG
KY429940  ---------- ---------- ---------- ---------- ---------- ----------
KY499716  ---------- ---------- ---------- ---------- ---------- ----------
          1381                                                        1440
KY429941  GTATATTACT AAATATCTCA ATATATAAGC CTGTGACTCT TAATAGTATT TCCCCTGCAC
KY429940  ---------- ---------- -------AGC CTGTGACTCT TAATAGTATT TCCCCTGCAC
KY499716  ---------- ---------- -------AGC CTGTGACTCT CAATAGTATT TCCCCTGCAC
          1441                                                        1500
KY429941  ATGTTTTACA CAATATTTGT TAACACATTA GACCACCACC GAAAAAATCC TTGTTTCCAT
KY429940  ATGTTTTACA CAATATTTGT TAACACATTA GACCACCACC GAAAAAATCC TTGTTTCCAT
KY499716  ATGTTTTACA CAATATTTGT TAACACATTA GACCACCACC GAAAAAATCC TTGTTTCCAT
          1501                                                        1560
KY429941  GCTTTCCCTC TATGTGAAAT TGTTTGTTTA CACACGCAAA CACACAATCA GCCTGAAAAA
KY429940  GCTTTCCCTC TATGTGAAAT TGTTTGTTTA CACACGCAAA CACACAATCA GCCTGAAAAA
KY499716  GCTTTCCCTC TATGTGAAAT TGTTTGTTTA CACACGCAAA CACACAATCA GCCTGAAAAA
          1561                                                        1620
KY429941  TGAAAGAAAG AAAAATCAAT GTCTGCACTT ACGTGAGTCC CTCGATCTTA TAGGTCATGT
KY429940  TGAAAGAAAG AAAAATCAAT GTCTGCACTT ACGTGAGTCC CTCGATCTTA TAGGTCATGT
KY499716  TGAAAGAAAG AAAAATCAAT GTCTGCACTT ACGTGAGTCC CTCGATCTTA TAGGTCATGT
          1621                                                        1680
KY429941  CATGGTTTGA AAAGATCTGC AAATTGTGAA AAGGAAACAT AAGAATGAAT ACCACTTAAA
KY429940  CATGGTTTGA AAAGATCTGC AAATTGTGAA AAGGAAACAT AAGAATGAAT ACCACTTAAA
KY499716  CATGGTTTGA AAAGATCTGC AAATTGTGAA AAGGAAACAT AAGAATGAAT ACCACTTAAA
          1681                                                        1740
KY429941  ACGAGCAACC AAATCAAGGA GAAAAACTTC CCCATCCCCG TTTCACTGCC CCATCTCTCT
KY429940  ACGAGCAACC AAATCAAGGA GAAAAACTTC CCCATCCCCG TTTCACTGCC CCATCTCTCT
```

```
KY499716  ACGAGCAACC AAATCAAGGA GAAAAACTTC CCCATCCCCG TTTCACTGCC CCATCTCTCT
          1741                                                    1800
KY429941  ATTTCTCTCC TCCTGTCACC TATCAGATGA GGAGATAGAT AGAAAGAGGG GGACGATGAA
KY429940  ATTTCTCTCC TCCTGTCACC TATCAGATGA GGAGATAGAT AGAAAGAGGG GGACGATGAA
KY499716  ATTTCTCTCC TCCTGTCACC TATCAGATGA GGAGATAGAT AGAAAGAGGG GGACGATGAA
          1801                                                    1860
KY429941  ACAGGGATGG GGAAGTGTTT TATGTATATA TTACATACAT CCGCCATACG GGTCTGAAAC
KY429940  ACAGGGATGG GGAAGTGTTT TATGTATATA TTACATACAT CCGCCATACG GGTCTGAAAC
KY499716  ACAGGGATGG GGAAGTGTTT TATGTATATA TTACATACAT CCGCCATACG GGTCTGAAAC
          1861                                                    1920
KY429941  ACTTCAGCTA CATCAGCGCC CATCTTTGTA TCATAAAACT CTTTCTTGTA AATGCCTTCT
KY429940  ACTTCAGCTA CATCAGCGCC CATCTTTGTA TCATAAAACT CTTTCTTGTA AATGCCTTCT
KY499716  ACTTCAGCTA CATCAGCGCC CATCTTTGTA TCATAAAACT CTTTCTTGTA AATGCCTTCT
          1921                                                    1980
KY429941  TTAGGGACTT CAGGATCAAT TTGAAATGGA TGCCATCTGA GCTCAAACTC GTATCGATCA
KY429940  TTAGGGACTT CAGGATCAAT TTGAAATGGA TGCCATCTGA GCTCAAACTC GTATCGATCA
KY499716  TTAGGGACTT CAGGATCAAT TTGAAATGGA TGCCATCTGA GCTCAAACTC GTATCGATCA
          1981                                                    2040
KY429941  TTACCTTCCA CTAGAGCTTT GTCAAGGTTT CTTTTGCCCA CAAAGCACCA TGGACACACA
KY429940  TTACCTTCCA CTAGAGCTTT GTCAAGGTTT CTTTTGCCCA CAAAGCACCA TGGACACACA
KY499716  TTACCTTCCA CTAGAGCTTT GTCAAGGTTT CTTTTGCCCA CAAAGCACCA TGGACACACA
          2041                                                    2100
KY429941  GTGTCTGCAA TTATATCAAT TTCCACAAGC TTTTTCTTGA TTCCAGCCAT ATCTGCAACT
KY429940  GTGTCTGCAA TTATATCAAT TTCCACAAGC TTTTTCTTGA TTCCAGCCAT ATCTGCAACT
KY499716  GTGTCTGCAA TTATATCAAT TTCCACAAGC TTTTTCTTGA TTCCAGCCAT
          2101                                                    2160
KY429941  GTATCCCGAC AATATTTAAC ACTCAAACTA GATGGTGAAA TTGGCACTCA AACAATTGCT
KY429940  GTATCCCGAC AATATTTAAC ACTCAAACTA GATGGTGAAA TTGGCACTCA AACAATTGCT
KY499716
          2161                                                    2220
KY429941  GCCAATATGG CAACCATGAC ATATATTAGA GCCACCAAAT TCATCAAATC ATATATACAT
KY429940  GCCAATATGG CAACCATGAC ATATATTAGA GCCACCAAAT TCATCAAATC ATATATACAT
KY499716
          2221                                  2259
KY429941  TTATATCTAT ATTAACTGTT TTAATGTCAA CCCAACCGT
KY429940  TTATATCTAT ATTAACTGTT TTAATGTCAA CCCAACCGT
KY499716
```

Primer design ParMDO

CATCTAGTTTGAGTGTTAAATATTGTCGGGATACAGTTGCAGATATGGCTGGAATCAAGAAAAAGCTTGT
GGAAATTGATATAATTGCAGACACTGTGTGTCCATGGTGCTTTGTGGGCAAAAGAAACCTTGACAAAGCT
CTAGTGGAAGGTAATGATCGATACGAGTTTGAGCTCAGATGGCATCCATTTCAAATTGATCCTGAAGTCC
CTAAAGAAGGCATTTACAAGAAAGAGTTTTATGATACAAAGATGGGCGCTGATGTAGCTGAAGTGTTTCA
GACCCGTATGGCGGATGTATGTAATATATACATAAAACACTTCCCTATCCTGTTTTATCGTCCCCCTCTT
TCTATCTCCTCATCTGATAGGTGACAGGAGGAGAAAAATAGAGAGATGGGGAAGTTTTTCTCCTTGATTT
GGTTGCTCGTTTTAAGTGGTATTCATTCTTATGTTTCCTTTTCACAATTTGCAGATCTTTTCAAACCATG
ACATGACCTATAAGATCGAGGGACTCACGTAAGTGCAGACATTGATTTTTCTTTCTTTCATTTTTCAGGC
TGATTGTGTGTTTGCGTGTGTAAACAAACAATTTCACATAGAGGGAAAGCATGGAAACAAGGATTTTTTC
GGTGGTGGTCTAATGTGTTAACAAATATTGTGTAAAACATGTGCAGGGGAAATACTATTGAGAGTCACAG
GCTTATATATTGAGATATTTAGTAATATACCCATTCTTAGCACTAAAACTATAAATAAACCCTACATCAT
TTCATTTTTATAAACATACCCAAAAAAAACCCAAAAAATGACAAATTGTACTATTAAATTTAATTTTAAT
TATTAAATTACTTTGATACCCTATTGAGTGTTTTGGGTTTTTTTATGAAATTTTGGGTTGGGTTTGTTTT
AAGAAATCAATGGCAATTTTGTAATTTAAAAGAAGTTAAAAGCTTTTTTGTTATGTTATAAATGGGCTTT
GGGTGTATTTATAAATTCCATTTCATATAGGGTTTTTTTATAATTTGGGCTCATATATTGGGTATATTAG
TGAATCTCCCATATATATTTTGCTGGGCTACAGGATCATGATAAGCAGCATGATCTAGTGGATGAGATCT

GTCTTGGATATTTCACAGATGGGAGATTCATTGGGGACAGGTAAGATTGCATATGGTGATTTATTCTCTT
GTTTAGTTTTGTAACATGGGGAGGATTGATTAATCTATTCTTAAAATTCAAGCAATGGCTATGGATGCAA
AGATATGTTCTGGGTTAAGTACTTTTACATCCTTAATCTAATTTAAAACTTAAAAGAGGCACAAACATTT
GCAATTTGGGTGCAGGGATTATCTTCTGAAATGTGCTGAAAAGATTGAAATAGAAGGGGCAGCAGAGTTT
CTTGATGACCCCAACAATGGGCTCACTGAGGTCTGTTCTTCACCAATTCCTTCATGTTCCTATTATTTTC
CAGGCCAAGCTATGTTGAAGGACAGCAAAAACCAGATAGAGGATTGTGGAATTTTTGTTATGATCCTTTG
GTTCCAAAACAGGCCAAATTAAAACTAGAATCCTGCAACAGTCATTCCTTTCTCAGAAATAAACCAAACC
CCAAACTTTCTTCTATATATTTTTGAATGGAACTGACCTCGCTCATTATGATGCAATGAAGGTCAAGGAA
GAGCTTAAGAAGTACTCAGAAGTAAAAGGAATCCCATATTTTGTGGTGGGTGATGAAAACTCA
